# The Use of Rhythmic Auditory Stimulation to Optimize Treadmill Training for Stroke Patients: A Randomized Controlled Trial

**DOI:** 10.3389/fneur.2018.00755

**Published:** 2018-09-14

**Authors:** Stefan Mainka, Jörg Wissel, Heinz Völler, Stefan Evers

**Affiliations:** ^1^Neurological Specialist Hospital for Movement Disorders/Parkinson, Beelitz-Heilstätten, Germany; ^2^Department of Neurology, Vivantes Hospital Spandau, Berlin, Germany; ^3^Center of Rehabilitation Research, University of Potsdam, Potsdam, Germany; ^4^Department of Neurology, Lindenbrunn Hospital, Coppenbrügge, Germany

**Keywords:** stroke rehabilitation, exercise movement techniques, music therapy, music, gait

## Abstract

The use of functional music in gait training termed rhythmic auditory stimulation (RAS) and treadmill training (TT) have both been shown to be effective in stroke patients (SP). The combination of RAS and treadmill training (RAS-TT) has not been clinically evaluated to date. The aim of the study was to evaluate the efficacy of RAS-TT on functional gait in SP. The protocol followed the design of an explorative study with a rater-blinded three arm prospective randomized controlled parallel group design. Forty-five independently walking SP with a hemiparesis of the lower limb or an unsafe and asymmetrical walking pattern were recruited. RAS-TT was carried out over 4 weeks with TT and neurodevelopmental treatment based on Bobath approach (NDT) serving as control interventions. For RAS-TT functional music was adjusted individually while walking on the treadmill. Pre and post-assessments consisted of the fast gait speed test (FGS), a gait analysis with the locometre (LOC), 3 min walking time test (3MWT), and an instrumental evaluation of balance (IEB). Raters were blinded to group assignments. An analysis of covariance (ANCOVA) was performed with affiliated measures from pre-assessment and time between stroke and start of study as covariates. Thirty-five participants (mean age 63.6 ± 8.6 years, mean time between stroke and start of study 42.1 ± 23.7 days) completed the study (11 RAS-TT, 13 TT, 11 NDT). Significant group differences occurred in the FGS for adjusted post-measures in gait velocity [*F*_(2, 34)_ = 3.864, *p* = 0.032; partial η^2^ = 0.205] and cadence [*F*_(2, 34)_ = 7.656, *p* = 0.002; partial η^2^ = 0.338]. Group contrasts showed significantly higher values for RAS-TT. Stride length results did not vary between the groups. LOC, 3MWT, and IEB did not indicate group differences. One patient was withdrawn from TT because of pain in one arm. The study provides first evidence for a higher efficacy of RAS-TT in comparison to the standard approaches TT and NDT in restoring functional gait in SP. The results support the implementation of functional music in neurological gait rehabilitation and its use in combination with treadmill training.

**Clinical Trial Registration**: https://www.drks.de/drks_web/, identifier DRKS00014603

## Introduction

About 60% of all stroke patients (SP) have difficulties with walking (1). These are often caused by hemiparesis and/or sensory deficits of the lower extremity and/or trunk and are also due to uncoordinated movements. In addition to motor and sensory dysfunctions, symptoms such as spasticity, somato-sensory neglect, and cognitive malfunctioning may further impede walking. Thus, the restoration of gait is often a key focus of rehabilitation efforts, enhancing not only physical activity but also autonomy and participation in everyday life (2, 3).

Treadmill training (TT) with and without body weight support has been shown to improve functional gait in stroke patients effectively. A meta-analysis comparing 44 trials (*n* = 2,658 patients) revealed clear therapeutic effects on gait velocity and walking endurance, the latter only for TT with body weight support (1). However, the improvements were identified only for independent walkers while patients who walked with assistance did not show an additional benefit from TT (1). Lee's work (4) provided evidence that TT with a high walking velocity at the beginning of training is more effective when compared to a stepwise increase in velocity.

Rhythmic-auditory stimulation (RAS) is defined as a therapeutic application of pulsed rhythmic or musical stimulation in order to improve gait or gait related aspects of movement (5). It has been demonstrated that SP are able to synchronize their gait pattern to auditory stimulation using music with an embedded metronome (6–8). This led to immediate improvements in stride time and stride length symmetry as well as weight bearing time on the paretic side, while EMG showed a more balanced muscular activation pattern between the paretic and non-paretic sides (6). Training effects of RAS for SP were confirmed in a meta-analysis comparing 7 randomized controlled studies (*n* = 197) that showed improvements in functional gait performance (velocity, cadence, and stride length) (9). This work also gave evidence, that a musical stimulation is more effective in improving gait velocity and cadence then the metronome (9). Hayden et al. found that RAS became more effective when it is implemented earlier in the rehabilitation program. This provides evidence that the variation in time of the RAS-training during the rehabilitation process may affect the success of the treatment (10). The application of RAS on the treadmill (RAS-TT) was evaluated over a 3-week training period by Park et al. In that study metronome stimulation was used for 9 patients with chronic stroke. The results were compared with a group of 10 patients performing over ground RAS walking training (11). The RAS-TT group experienced greater improvements in gait velocity (11).

While RAS and TT have proven to be effective for gait training in SP, the efficacy of its combination (RAS-TT) in the early course of rehabilitation in SP has not been investigated to date. Therefore, we hypothesized that RAS-TT in the early course of rehabilitation would improve the clinical efficacy of TT for SP. The purpose of the present study was to investigate the functional improvements of gait using a rehabilitation therapy combining RAS and TT in order to assess its clinical efficacy for patients suffering the aftermaths of a stroke.

## Materials and methods

### Design

The study protocol was approved by the state authorization association for medical issues in Brandenburg, that determined on the 21st of January 2010 that no formal ethics approval was required. Patients gave their informed consent according to the Helsinki declaration.

The study was designed as a prospective, single center three arm clinical study with parallel groups. We enrolled patients who performed either RAS on the treadmill (RAS-TT) or treadmill training alone (TT). A third group that received neurodevelopmental treatment following the Bobath approach (NDT) served as a control group. The patients were randomly assigned to the three training interventions by a person not involved in the study using a block randomization (software randlist). Allocations were placed in sealed sequentially numbered envelopes and were not opened until the actual study inclusion. Thus, the patients, the responsible doctor, the assessing physiotherapist, and study manager were not informed beforehand regarding the group assignment.

We included stroke patients with a hemiparesis of the lower limb (at least 1 muscle group with muscle strength grade <5 as defined by the British Medical Research Council) or with an unsafe and asymmetrical walking pattern (by assessment of a physiotherapist). The patients had to be able to walk independently with assistive devices if necessary for at least 3 min.

Criteria for exclusion were the following: significantly disturbed language perception (marked by either the Aachener Aphasietest or Token Test), cognitive impairment (Mini Mental Status Test <26), major depression or productive psychosis, adjustment disorder with a need for medical treatment, peripheral arterial occlusive disease with walking distance <100 m, and coronary heart disease (instable angina pectoris).

**Figure 1:**
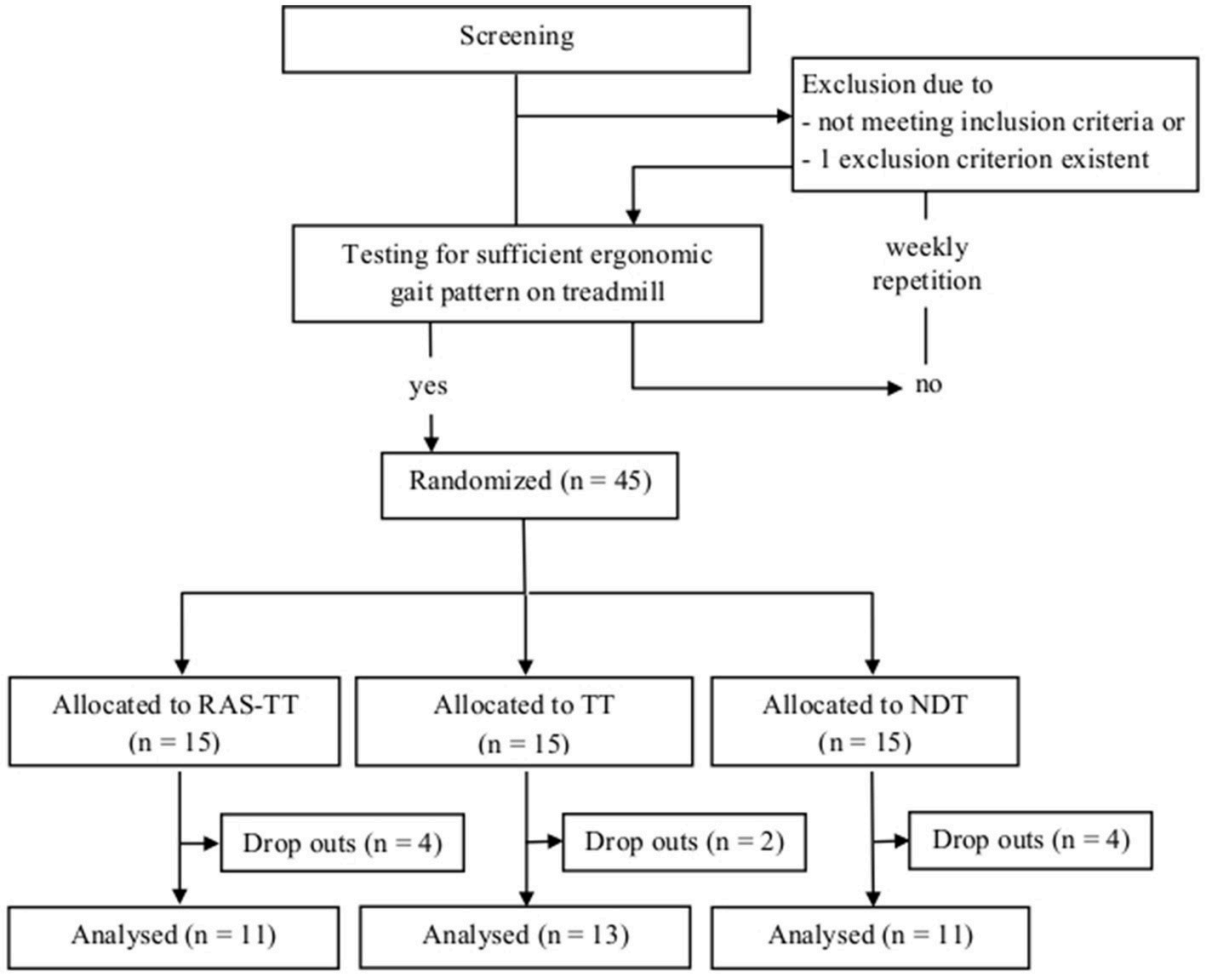
Patient flow chart of study design. RAS-TT, rhythmic auditory stimulation on treadmill; TT, treadmill training; NDT, neurodevelopmental treatment.

After having passed the diagnostics patients underwent a screening session on the treadmill. There they had to demonstrate a stable and sufficiently ergonomic gait. Candidates with insufficient quality of gait on the treadmill (multimodal neglect or spasticity as assessed by a physiotherapist) were postponed and re-screened every week (Figure 1).

### Subjects

Patients were recruited in a clinical center for neurological rehabilitation in Beelitz-Heilstaetten/Germany. We included 45 stroke patients, of which ten were excluded during the intervention phase due to the following reasons: five for administrative reasons (funding of the in-patient rehabilitation was stopped) distributed over all three groups (RAS-TT: 2, TT: 1, NDT: 2); four patients developed acute medical complications that were unrelated to the movement therapies (occurring in group RAS-TT: 2 and group NDT: 2); one patient reported pain in the paretic arm following treadmill training (TT), which seemed to be associated with increasing spasticity. The data of 35 patients were used for data analysis (Table 1).

**Table 1:** Subject data with location of stroke and use of assistive device.

	**RAS-TT**	**TT**	**NDT**
Number	11	13	11
Age (years)	63.7 ± 8.8	65.5 ± 8.5	61.1 ± 8.6
Gender (F/M)	4/7	2/11	3/8
Time between stroke and start of study (days)	42.6 ± 30.1	46.9 ± 23.3	36.0 ± 16.7
Side of Lesion (L/R)	6/5	4/9	5/6
Location of stroke			
Middle cerebral artery	5	6	6
Brain stem	3	5	2
Basal ganglia/Thalamus	2	1	1
Internal capsule	–	1	1
Anterior cerebral artery	1	–	–
Posterior cerebral artery	–	–	1
Use of assistive device			
None	5	8	9
Walking aid	5	4	1
Ankle-foot orthosis + walking aid	1	1	1

*RAS-TT, rhythmic auditory stimulation on treadmill; TT, treadmill training; NDT, neurodevelopmental treatment*.

### Training

The three interventions RAS-TT, TT and NDT were performed five times a week over of 4 weeks. All interventions were carried out as a single therapy with individual instruction from a physiotherapist.

In the NDT patients practiced walking on an even surface, stair stepping, handling of a walking aid (if necessary) based on their actual walking disability for 30 min. During this treatment movements were directed toward an enhanced activation of the paretic side while inhibiting an immoderate co-activation of the non-affected side. NDT was applied by physiotherapists who had received an advanced training in the NDT for neurologically impaired adults.

No body weight support or inclination was used for the RAS-TT and the TT interventions. Patients walked on the treadmill (Loko S 70, Woodway, Waukesha/USA) holding themselves with at least one hand at the side bar. Therapists were instructed to keep the treadmill pace at a maximum level while striving for a normalized physiological gait pattern (e.g., limitation of compensational movements and reduction of pathological muscle tone). The training time was increased continually (15 min in week 1; 17 min in week 2; 20 min in weeks 3 and 4). Therapists were allowed to set breaks for the patient when needed.

For the RAS-TT intervention, patients listened to functional training music via ear plugs through an ordinary MP3 player while walking on the treadmill. The music was designed (software cubase 3 SE) according to the criteria described by Thaut et al. (5): clearly structured rhythm with strongly accentuated meter, adequate volume with no significant perturbations or qualitative changes in intensity, familiar melody, no lyrics, and a superimposed salient high-pitch bell sound to foster the metered walking pace additionally [(12), Audio 1]. The beat rate of the music was set to match the patients' cadence on the treadmill. Once audio-motor coupling was achieved, the musical tempo was slowed down a little in order to induce greater step lengths. The RAS-TT therapists were continually supervised by a certified neurologic music therapist. Therapists were assigned to one intervention type only to avoid a bias caused by preference for a certain intervention.

All patients were given extra conventional physiotherapy treatment either 30 or 60 min per week according to the level of impairment, which was constantly updated. All patients performed further training therapies according to their medical needs (e.g., occupational therapy, speech therapy, neuropsychological treatment, sports therapy, physical therapy applications, and others).

### Assessment

Our primary outcome parameters were gait velocity, cadence and stride length. Secondary outcome parameters were gait symmetry, endurance and postural stability. Assessments were carried out by physiotherapists blinded for the group assignment. Pre-assessments were carried out at the beginning of the training period. Post-testing was performed on the day after completing the 4 weeks training.

We included a fast gait speed test (FGS), a gait analysis with the locometre (LOC), a 3-min walking test (3MWT), and an instrumental evaluation of balance (IEB).

In the FGS, patients were asked to walk safely as fast as possible toward a goal, while starting 2 meters ahead of the start mark. The time was measured with a stop watch, and the heel strikes within a marked 10 meter walk way were counted. Patients were allowed walking aids when they needed them and were asked to use them again for the post-test. The test was performed twice. For the statistical analysis the data from the second trial were used. Stride length, gait velocity and cadence were calculated using the formula presented by Flansbjer et al. (13). The LOC represents an apparatus based analysis of walking performed with the locometre (SATEL, Blagnac, France) according to Bessou et al. (14). With this device, the longitudinal displacements of the feet are transmitted via threads to a pulley connected to an optical length-voltage transducer. Record analysis provides quantitative data to determine side related step length, cycle time and stance time, as well as walking velocity, cadence and stride length, recorded over a distance of 7 meters. The test was carried out twice at maximum walking speed. The data collected from the second trial were used for the statistical analysis.

In the 3MWT patients were asked to walk for 3 min as far as they could. The distance covered was measured in meters. The IEB was performed using a force platform (SATEL, Blagnac, France). In this test, postural balance was evaluated in a standing position on a firm surface with eyes open. The duration of recording was 51 s (sampling rate: 40 Hz). The length of sway, sway area and mean lateral displacement of the center of pressure were calculated (15).

### Data analysis

For the calculation of the sample size an ANOVA with repeated measures for the time effect was conducted (software G * power 3.0.10) with an error probability of α = 0.05, a given power of 0.8. The required sample size was 36 for the intended effect size f = 0.25.

Due to the applied power analysis we decided to enroll a total sample of 45. As some of the data were not normally distributed and there was no fitting non-parametric statistical method for the study design, an analysis of covariance (ANCOVA) was run (SPSS 11.5). Thus, post-intervention assessment measures were adjusted for the two covariates: (1) respective pre-intervention measure and (2) the time between the stroke and the start of training intervention. Significant pre-post-effects were followed up group-wise using Bonferoni corrected *t*-tests with a level of significance of *p* = 0.0167. The ANCOVA was calculated on the basis of the following comparisons of covariates and post-intervention measurements for each intervention type: the linear relationship between covariates and post-intervention measures, which was assessed by visual inspection of a scatterplot, the homogeneity of regression by determining the interaction term, the normal distribution of residuals using Shapiro-Wilk's test and the homoscedasticity and homogeneity of variances using visual inspection of a scatterplot and Levene's test of homogeneity of variance. Statistical outliers were detected by identifying cases with standardized residuals >±3. The assumptions for ANCOVA were met for all assessment parameters of the LOC, the FGS, and the 3MWT. One outlier in the standardized residuals (as defined by being greater than the standard deviation ±3) was found in the LOC parameter stride length. This outlier was kept for the analysis.

## Results

There were no significant differences between the groups with respect to baseline characteristics (i.e., age, gender), except for the use of assistive devices as these were notably less present in the NDT group. The groups differed also in time between stroke and study inclusion.

**Figure 2:**
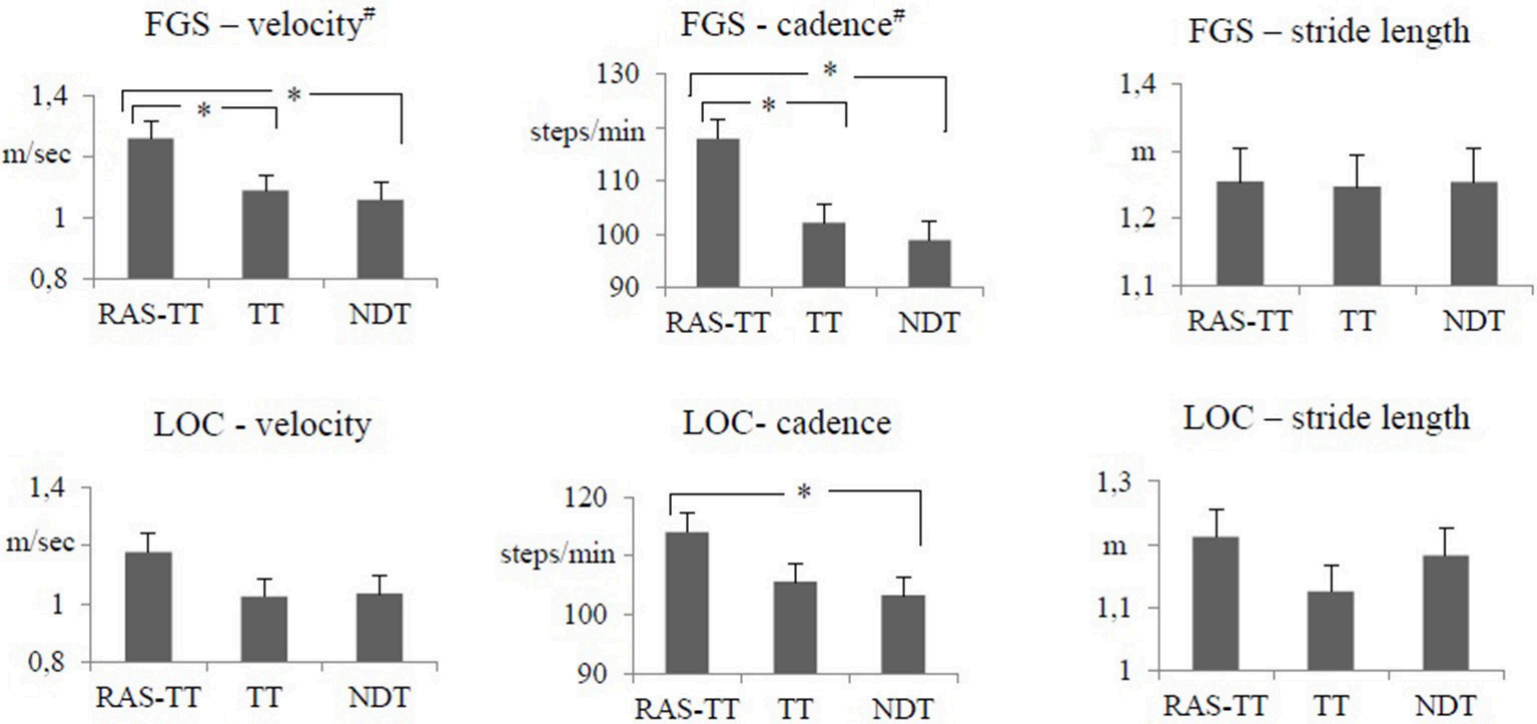
Primary outcome measures: adjusted means of post intervention gait parameters with pre-intervention measures and time between stroke and study entrance as co-varieties. Error bars indicate the standard error. ^#^Statistically significant group difference. ^*^Statistically significant group contrast. (*p* < 0.05). RAS-TT, rhythmic auditory stimulation with treadmill training; TT, treadmill training; NDT, neurodevelopmental treatment; FGS, fast gait speed test; LOC, gait analysis with locometre.

The results of the FGS showed significant time effects for the parameters gait velocity, cadence and stride length. For RAS-TT, we found significant pre-post-changes that corresponded with moderate to strong effect sizes (velocity: *p* < 0.001; cadence: *p* = 0.001; stride length: *p* < 0.001). For TT, there were significant changes in velocity (*p* < 0.007) and stride length (*p* < 0.001) while cadence did not improve significantly (*p* = 0.283). In the NDT group, no significant changes were observed (velocity: *p* = 0.029; cadence: *p* = 0.93; stride length: *p* = 0.018, Table 2). The ANCOVA showed statistically significant differences between groups for gait velocity [*F*_(2, 34)_ = 3.864, *p* = 0.032, partial η^2^ = 0.205] and for cadence [*F*_(2, 34)_ = 7.656, *p* = 0.002, partial η^2^ = 0.338]. The adjusted post-values compared by group contrasts were significantly higher in favor of RAS-TT (velocity: RAS-TT to TT *p* = 0.031, RAS-TT to NDT *p* = 0.017; cadence: RAS-TT to TT *p* = 0.004, RAS-TT to NDT *p* = 0.001) (Figure 2). The outcomes in the FGS stride length measures did not show any group differences.

**Table 2:** Pre and post-intervention measures and effect sizes for gait and postural balance parameters (means ± standard deviation with 95% confidence interval).

**Parameter**	**RAS-TT (*****N*** = **11)**			**TT (*****N*** = **13)**			***NDT (N** = **11)***		
	**Pre**	**Post**	***d***	**Pre**	**Post**	***d***	**Pre**	**Post**	***d***
**FAST GAIT SPEED TEST**									
Velocity [m/s]^†^	0.92 ± 0.46	1.27 ± 0.48^*^	0.75	0.85 ± 0.36	1.03 ± 0.35^*^	0.50	0.95 ± 0.37	1.12 ± 0.40	0.43
Cadence [steps/min]^†^	96.7 ± 22.8	116.5 ± 22.5^*^	0.87	98.0 ± 19.3	101.5 ± 16.8	0.19	101.4 ± 21.31	101.0 ± 16.9	−0.02
Stride length [m]^†^	1.09 ± 0.35	1.28 ± 0.32^*^	0.57	1.01 ± 0.28	1.19 ± 0.28^*^	0.66	1.10 ± 0.27	1.30 ± 0.34	0.65
**LOCOMETRE GAIT ANALYSIS**									
Velocity [m/s]^†^	0.84 ± 0.43	1.20 ± 0.47^*^	0.81	0.76 ± 0.29	0.97 ± 0.34^*^	0.69	0.84 ± 0.39	1.07 ± 0.36	0.60
Cadence [steps/min]^†^	96.6 ± 25.0	115.0 ± 23.4^*^	0.76	91.2 ± 19.6	102.7 ± 15.3^*^	0.65	98.4 ± 23.7	105.7 ± 17.7	0.35
Stride length [m]^†^	0.99 ± 0.31	1.22 ± 0.31^*^	0.76	0.96 ± 0.26	1.12 ± 0.29^*^	0.55	0.97 ± 0.29	1.18 ± 0.28^*^	0.74
**3 MIN WALKING TIME TEST**									
Distance [m]^†^	162.2 ± 69.4	216.8 ± 75.5^*^	0.75	146.5 ± 62.0	170.5 ± 53.7	0.41	180.3 ± 108.4	218.3 ± 119.9^*^	0.33
**INSTRUMENTAL EVALUATION OF BALANCE**									
Mean lateral COP displacement [mm]	11.2 ± 9.5	11.6 ± 9.3	0.05	15.9 ± 10.7	13.4 ± 10.6	−0.23	15.3 ± 9.9	13.0 ± 10.5	−0.23
Length of COP sway [mm]^†^	714.2 ± 393.5	702.5 ± 525.0	−0.03	938.6 ± 486.5	834.9 ± 410.9	−0.23	722.6 ± 274.7	632.6 ± 147.5	−0.41
Sway area of COP [mm^2^]	485.6 ± 602.9	397.8 ± 364.9	−0.18	450.1 ± 245.1	351.5 ± 181.7	−0.48	326.6 ± 216.3	259.9 ± 147.5	−0.36

† *Statistically significant time effect (p < 0.05)*,

* *statistically significant differences between pre and post-intervention values (p < 0.01$\bar{6}$), d*,

*effect size Cohen's; 
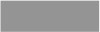
, strong effect; 
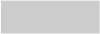
, moderate effect; 
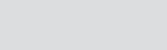
, small effect; RAS-TT, rhythmic auditory stimulation on treadmill; TT, treadmill training; NDT, neurodevelopmental treatment; COP, center of pressure*.

The LOC allowed us to look at the lower extremities separately. Thereby, the RAS-TT patients improved significantly in 3 out of 6 spatial and temporal parameters, while TT patients showed significant changes in two parameters and NDT patients in no parameter. RAS-TT showed higher effect sizes representing moderate to strong effects throughout (Table 3). Also in the LOC measures for gait velocity, cadence, and stride length we observed statistically significant time effects. Looking at pre-post-effects in the three therapeutic interventions, significant changes were found for RAS-TT and TT throughout (velocity and stride length: *p* < 0.001, cadence for RAS-TT: *p* = 0.001, for TT *p* = 0.002) with moderate effect sizes. The results of NDT showed a significant improvement in stride length only (*p* = 0.006) while velocity (*p* = 0.017) and cadence changes (*p* = 0.102) did not reach the level of significance (Table 2). There were no statistically significant differences in adjusted post-intervention gait parameters between the three groups [for velocity: *F*_(2, 34)_ = 1.861, *p* = 0.173, partial η^2^ = 0.11; for stride length: *F*_(2, 34)_ = 1.108, *p* = 0.343, partial η^2^ = 0.069]. The analysis for cadence fell short of the level of significance with *F*_(2, 34)_ = 3.242, *p* = 0.053, partial η^2^ = 0.178. Group contrasts here revealed a significant difference between RAS-TT and NDT (*p* = 0.023) while the contrast RAS-TT to TT failed to show significance (*p* = 0.06) (Figure 2).

**Table 3:** Spatial and temporal parameters in pre and post-assessment and effect sizes for the impaired and the unimpaired lower extremity from gait analysis with locometre (means ± standard deviation with 95% confidence interval).

**Parameter**	**RAS-TT (*****N*** = **11)**			**TT (*****N*** = **13)**			**NDT (*****N*** = **11)**		
	**Pre**	**Post**	***d***	**Pre**	**Post**	***d***	**Pre**	**Post**	***d***
**IMPAIRED LOWER EXTREMITY**									
Step length [m]^†^	0.49 ± 0.19	0.60 ± 0.22	0.556	0.47 ± 0.15	0.54 ± 0.16	0.403	0.48 ± 0.19	0.59 ± 0.13	0.665
Cylce time [s] ^†^	1.32 ± 0.33	1.08 ± 0.22^*^	−0.824	1.40 ± 0.43	1.20 ± 0.21	−0.623	1.30 ± 0.42	1.17 ± 0.27	−0.365
Stance phase portion [%] ^†^	61.47 ± 4.47	59.16 ± 4.08	−0.538	59.92 ± 4.92	59.88 ± 3.66	−0.008	63.17 ± 8.04	58.76 ± 4.92	−0.662
**UNIMPAIRED LOWER EXTREMITY**									
Step length [m]^†^	0.50 ± 0.16	0.62 ± 0.21	0.647	0.49 ± 0.16	0.58 ± 0.15^*^	0.63	0.50 ± 0.17	0.60 ± 0.17	0.605
Cylce time [s]^†^	1.32 ± 0.33	1.08 ± 0.22^*^	−0.834	1.38 ± 0.37	1.20 ± 0.20^*^	−0.63	1.31 ± 0.43	1.18 ± 0.27	−0.378
Stance phase portion [%]^†^	65.63 ± 6.63	60.29 ± 5.94^*^	−0.848	64.57 ± 5.88	62.15 ± 5.55	−0.423	64.57 ± 7.21	61.76 ± 4.82	−0.458

† *Statistically significant time effect (p < 0.05)*,

* *statistically significant differences between pre and post-intervention values (p < 0.01$\bar{6}$), d*,

*effect size Cohen's; 
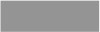
, strong effect; 
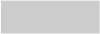
, moderate effect; 
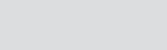
, small effect; RAS-TT, rhythmic auditory stimulation on treadmill; TT, treadmill training; NDT, neurodevelopmental treatment*.

**Figure 3:**
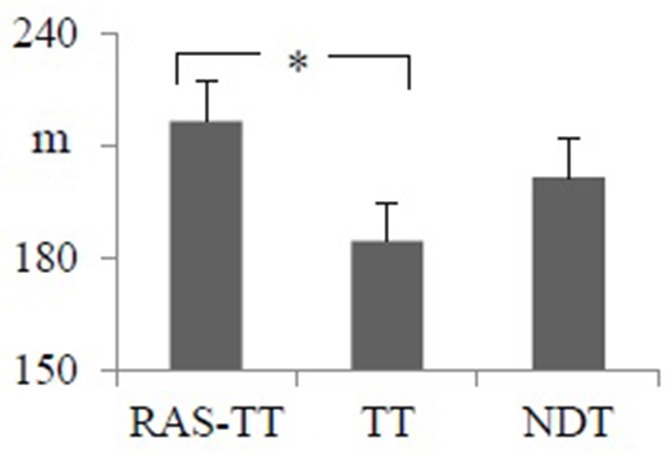
Adjusted means of post intervention walking distance from the 3 min walking time test with pre-intervention measures and time between stroke and study entrance as co-variates. Error bars indicate the standard error. ^*^Statistically significant group contrast. (*p* < 0.05). RAS-TT, rhythmic auditory stimulation with treadmill; TT, treadmill training; NDT, neurodevelopment treatment.

Walking endurance as assessed by the 3MWT improved significantly across all groups. The RAS-TT and NDT group experienced significant pre-post-changes (RAS-TT: *p* < 0.001, d = 0.75; NDT: *p* = 0.001, *d* = 0.75) while patients in the TT group did not improve significantly (*p* = 0.046) despite a small effect size of *d* = 0.413. The group comparison with the regression analysis showed no significance [*F*_(2, 34)_ = 2.434, *p* = 0.104, partial η^2^ = 0.136] but revealed a significant group contrast between RAS-TT and TT (*p* = 0.033) (Table 2 and Figure 3).

In the IEB, only the parameter length of sway showed a significant time effect (*p* = 0.048). No significant pre-post-improvements were present when looking separately at the three groups.

## Discussion

The objective of this study was to evaluate whether the focused use of functional music can make TT for SP more effective. In our study, this led to significantly improved gait velocity and cadence in the FGS. Single leg spatio-temporal parameters also showed higher effect sizes in this group. The latter can be stated as a hint for an optimized kinematic gait pattern, due to audio-motor coupling. The gains in velocity and cadence were not confirmed in the apparatus based gait analysis, where cadence only showed a tendency for higher values in favor of the RAS-TT group. The very high reliability of the FGS test for hemiparetic stroke patients has been proven with an intra-class-correlation of 0.97 (13, 16). For the apparatus based gait analysis LOC, there has been only limited evaluation of quality criteria assessing a small sample (*n* = 12) at comfortable walking speed (ICC 0.93) (17), but not for walking at maximum speed, which was assessed in our study. Nonetheless, we have included that assessment in order to evaluate kinematic changes for both lower extremities separately. It is possible that the patients felt disturbed by the straps attached to their feet when trying to reach maximum speed rather suddenly. In sum the results support our hypothesis that TT with auditory stimulation from functional music leads to greater improvement in functional gait. However, this was not reproduced in the gait endurance test, where all patients improved equally well.

Our balance measures showed a significant time effect on the length of sway of COP. The fact, that this improvement was distributed equally across groups while gait improvement was not could indicate that balance functions improve unrelated to special training interventions due to general recovery from sensorimotor deficits. In order to deepen the understanding of the role of movement training in the improvement of balance functions in this clientele, further investigation using balance measures as an inclusion criteria should be carried out.

In conventional TT, the therapist mainly intervenes by adjusting the pace of the treadmill. Thus, the speed of gait can be regulated. The additional use of music enables the inherent correction of step frequency and gait pattern, by using auditory motor coupling. RAS has been used so far to increase cadence in gait training on the ground. Typically, cadence in TT with stroke patients is almost as high as in walking on the ground while stride length is short (18). This is attributed to negative psychological reactions such as anxiety and insecurity (18). In our RAS-TT approach, we used the music to induce bigger steps by lowering the musical tempo in order to normalize the hemiparetic gait pattern and to reduce motoric and psychological stress. Presumably, the extra benefit of the RAS-TT patients can be attributed to an improved temporal stability that was induced via audio-motor entrainment through the adjusted music. It remains an open question whether a different manner of music adjustment would have led to different functional results.

In our study, there was one drop-out that was attributed to TT. In general, patients coped well with this form of apparatus based therapy while performing comparable to or better than when NDT was used. Even though we used computerized music, none of the patients would consider omitting the music. A few patients reported that they became bored with the long stereotype arrangement, but still felt the advantage for their gait training and wanted to carry on with it. According to our clinical observations, the severe motoric problems stroke patients experience call for the use of clear-cut and foreseeable music. This is especially important when training on the treadmill. Nonetheless, these considerations can be realized in a more refined and artistically ambitious manner. Ideally, patients will be able to choose their preferred musical genre in the future. This could lead to a still better compliance and to additional motivational effects.

Our study has some limitations. The power of the measured effects is limited by the small sample size. We were not able to follow up on patients. Therefore, it remains uncertain whether the effects would persist over a longer period of time. A weak point of our assessment battery can be seen in the absence of participation oriented assessments like the Barthel Index or functional ambulatory category.

Our data suggest, that RAS-TT can be considered a form of training that optimizes gait rehabilitation in stroke patients. The need for a larger study, preferably comparing TT, and RAS-TT, remains. Furthermore, it would be interesting to investigate, whether patients with either predominantly paretic or more sensory symptoms benefit most from the music intervention.

## Data availability statement

The raw data supporting the conclusions of this manuscript will be made available by the authors, without undue reservation, to any qualified researcher.

## Author contributions

SM conceptualized and directed this study, performed data analysis, wrote the first draft of the manuscript. JW contributed to the conception and design of the study. All authors contributed to manuscript revision, read and approved the submitted version.

### Conflict of interest statement

The authors declare that the research was conducted in the absence of any commercial or financial relationships that could be construed as a potential conflict of interest.

## Supplementary material

Audio 1 Functional music used for rhythmic auditory stimulation on the treadmill at 77 bpm.
